# A French–Greek Cross-Site Comparison Study of the Use of Automatic Video Analyses for the Assessment of Autonomy in Dementia Patients

**DOI:** 10.3390/bios10090103

**Published:** 2020-08-21

**Authors:** Anastasios Karakostas, Alexandra König, Carlos Fernando Crispim-Junior, François Bremond, Alexandre Derreumaux, Ioulietta Lazarou, Ioannis Kompatsiaris, Magda Tsolaki, Philippe Robert

**Affiliations:** 1 Information Technologies Institute, Centre for Research and Technology Hellas (CERTH-ITI), 57001 Thessaloniki, Greece; akarakos@iti.gr (A.K.); ikom@iti.gr (I.K.); 2 EA CoBTeK, University of Nice Sophia Antipolis, 06100 Nice, France; akonig03@gmail.com (A.K.); derreumaux.a@chu-nice.fr (A.D.); 3 Centre Mémoire de Ressources et de Recherche, CHU de Nice, 06100 Nice, France; 4 INRIA-STARS Team-Sophia Antipolis, CEDEX, 06902 Sophia Antipolis, France; carlos.crispim-junior@liris.cnrs.fr (C.F.C.-J.); francois.bremond@inria.fr (F.B.); phil.robert@gmail.com (P.R.); 5 1st Department of Neurology, Medical School, Aristotle University of Thessaloniki, 54124 Thessaloniki, Greece; tsolakim1@gmail.com; 6 Greek Association of Alzheimer’s Disease and Related Disorders, 54643 Thessaloniki, Greece

**Keywords:** smart home, dementia, ambient assisted living, assistive technology, sensors, remote monitoring

## Abstract

Background: At present, the assessment of autonomy in daily living activities, one of the key symptoms in Alzheimer’s disease (AD), involves clinical rating scales. Methods: In total, 109 participants were included. In particular, 11 participants during a pre-test in Nice, France, and 98 participants (27 AD, 38 mild cognitive impairment—MCI—and 33 healthy controls—HC) in Thessaloniki, Greece, carried out a standardized scenario consisting of several instrumental activities of daily living (IADLs), such as making a phone call or preparing a pillbox while being recorded. Data were processed by a platform of video signal analysis in order to extract kinematic parameters, detecting activities undertaken by the participant. Results: The video analysis data can be used to assess IADL task quality and provide clinicians with objective measurements of the patients’ performance. Furthermore, it reveals that the HC statistically significantly outperformed the MCI, which had better performance compared to the AD participants. Conclusions: Accurate activity recognition data for the analyses of the performance on IADL activities were obtained.

## 1. Introduction

Alzheimer’s Disease (AD) is an incurable, irreversible state which leads to a loss of autonomy in the activities of daily living, associated with a significant decrease in quality of life [1]. According to the 2016 estimation of Alzheimer’s Disease International (ADI) [2], 47 million people worldwide suffer from dementia today, and this number is expected to grow to 131 million by 2050. This neurodegenerative condition is characterized by the progressive deterioration of both cognitive and functional abilities, particularly in complex tasks [3,4,5,6]. Among other symptoms, such as apathy, withdrawal, and depression, the gradual loss of the ability to perform daily living activities (i.e., domestic activities, medication) is a major problem which needs to be addressed.

Recent studies have shown that deterioration in performing instrumental activities of daily living (IADLs) may be an early predictor for cognitive deterioration, and possibly even for conversion from mild cognitive impairment (MCI) to AD [7]. These particular findings are similar to previous results showing that the deterioration of the IADLs is affected by cognitive function, and relatively early in the dementia spectrum [8]. In particular, in the MCI [9,10], the executive functioning as part of specific IADL tasks requires frontal cortex activation [11]. More specifically, the evaluation of IADLs has recently gained more attention in clinical and neuroscience research studies and should be incorporated, not only as a part of the assessment for setting the diagnosis in dementia, but it should be important to measure the efficiency and the efficacy of a rehabilitation program [12,13]. However, to indicate the level of impairment in IADLs is controversial due to the absence of particular standards, both in the practical or theoretical definition [14]. Therefore, clinicians are based on typical neuropsychological assessment in order to provide symptom markers of AD that are important for setting the early diagnosis. Moreover, until now, the evaluation of IADL is mostly based on questionnaires and often rely on informants reports, such as the Disability Assessment for Dementia scale (DAD), or the IADL scale of Lawton and Brody [15]. These solutions suffer from biases and subjective misperceptions in informants [16,17] as well as the possibility that some older adults do not have an individual who can comment on the impact of cognitive impairment on their routine activities. In general, existing functional assessments lack sufficient sensitivity to detect subtle functional changes or differences in behavior [9]. This leads to the need for better measures of functional changes in people with the earliest changes related with AD [18].

It is widely known that the association between activities of daily living (ADLs) and AD has gained some research interest in recent decades [19]. In particular, neuroscientists are very interested in determining the functional activity of individuals, so as to gain a better understanding of the daily obstacles which negatively affect cognitive function. This information will also assist people in order to successfully complete daily activities while maintaining their independence. In detail, advanced clinical research has only recently started to seek for tools in order to assess the relation between cognitive function and ADLs so as to detect the subtle changes that could provide information for MCI or transitions from MCI to mild AD. The need of a more holistic, objective and immediate evaluation has paved the way to explore alternative ways of IADL assessment by developing new detailed caregiver-based computerized IADL tests [20] or direct tools based on patients’ performance [21]. They differ from the traditional questionnaires, such as the IADL Lawton scale, because both directly observe the person while performing particular IADLs (e.g., making a phone call or managing financial issues). Moreover, in a recent study, researchers have developed such direct performance-based assessment of people with (e.g., the functional living skills assessment—FLSA) with a focus on IADL and high-order social activity in daily life [22]. During their testing, a researcher is directly observing the participant while carrying out practical tasks.

Currently, the qualitative character of existing methods combined with biased evaluations points to a need for objective and systematic assessment tools for the objective and timely assessment of dementia development. The clinical expertise and literature review indicate that information communication technologies (ICTs) are not yet able to provide a direct diagnosis of AD and the related disorders, but can provide additional information for the assessment of specific domains (behavior, cognition, activities of daily living). Therefore, together with clinician and biological related information, ICTs can assist with detecting the early symptoms across AD and the related disorders. However, ICT-related assessment may hold some limitations, such as the need of the presence of researchers during the evaluation, and the stress of the patient due to an unfamiliar environment or due to the extensive time that may be required to perform all the activities [23].

ICTs, and in particular, automatic video analyses of patients carrying out various IADLs, could be an innovative assessment method to overcome the described limitations, reduce biases due to human interpretation and increase ecological value by completely removing the human observer from the assessment site. Several recent studies have employed pressure sensors, passive infrared sensors or wearable accelerometer to detect and assess ADL performance, achieving an accuracy of 96.5% [24]. Others have used video cameras, door sensors, wearable kinetic sensors and microphones to receive ADL-related data, which were processed by a support vector machine, achieving a sensitivity of 97.8% [25]. Such techniques and thus, our proposed automatized video-based IADL assessment, differ from current tools by enabling the patients’ performances and actions to be captured remotely in real-time and real-life situations, and being accurately evaluated in order to provide the clinician with objective performance measures and a “second opinion” regarding the overall state of functionality of the patient [26].

Based on the efforts (and their limitations) described above, we developed a framework (Dem@care framework), which was based on the ambient assisted living (AAL) approach and technologies, and it was applied in a lab environment during clinical intervention. In particular, the data coming from the sensor outputs and fused with machine learning algorithms can provide clinicians with a detailed and holistic profile about the ability of a patient to perform specific ADLs. In the previous work of members of the Dem@care project consortium, the use of such video sensor technology has been already validated for IADL assessment in 19 healthy subjects and 19 people with dementia [10,27]. Furthermore, it was demonstrated that autonomy assessment was approached as a classification task using artificial intelligence methods, that takes as input the parameters extracted by an event monitoring system, here referred to as behavioral data. Activities were recognized with high precision and the autonomy group classifier obtained a precision of 83.67% when combining the kinetic parameters extracted from physical tasks and IADLs [10].

The objective of this study was to investigate whether the same protocol of these studies with the same equipment transferred to another clinical site in another country and cultural context will provide similar results. From a clinical perspective, a functional measure that takes into account strategy use could increase the understanding of everyday difficulties experienced by elders. Moreover, the types of compensatory strategies used to support functional independence could also contribute in improving treatment recommendations and cognitive rehabilitation programs.

To compare the autonomy of people during the execution of the protocol at both sites, we employed a computer vision system to automatically recognize and summarize the activities performed by each participant. Three main evaluations were carried out: the analysis of the correlation between the patients’ activities that are automatically recognized by the employed system and the activities manually annotated by domain experts; the analysis of differences between the cognitive status of patients based on the activities carried out during the protocol; and finally, the analysis of the activity performances between sites. We hypothesized that performing activities with which the majority of people are familiar with could give us information about task-based difficulties, such as errors, confusion, and disorientation that an individual with cognitive impairment might experience while performing them. The ultimate goal of this study was to evaluate whether a computer vision system can become a complementary assessment method for the diagnosis of cognition, by automatically analyzing the ability of people with dementia in activities of daily living, overcoming clinical biases in different sites.

## 2. Materials and Methods

### 2.1. Study Participants and Clinical Assessment in Nice

The initial protocol to more objectively assess IADL functioning was designed by the team of the Memory clinic in Nice, in France, which served as a first test site for implementing the use of the Dem@care system in clinical practice, and was followed by a transfer to a second clinical site in Thessaloniki, in Greece.

Eleven (11) participants, aged 65 or above, were recruited within the Dem@care protocol at the Nice Memory Research Center located at the Geriatric department of the University Hospital. The study was approved by the local ethics committee of Nice and only participants with the capacity to consent to the study were included. Each participant gave informed consent before the first assessment. It was a non-randomized study involving two diagnosis groups of participants, patients with mild cognitive impairment (MCI) and healthy controls (HC). For the MCI group, the patients were diagnosed using the Petersen clinical criteria [28] and only people with a mini-mental state examination (MMSE) [29] with total score over 24 (mild dementia) were included in the study. Subjects were not included if they had a history of head trauma with a loss of consciousness, psychotic or aberrant motor activity (tremor, rigidity, Parkinsonism) as defined by [30] in order to control for any possible motor disorders influencing the ability to carry out IADLs.

Each participant underwent a standardized neuropsychological assessment with a psychologist. In addition, medical, clinical and demographical information were collected. The MMSE was administered in order to assess the global cognitive functioning of the participants. Moreover, in order to assess other cognitive functions, we administered the Free and Cued Selective Reminding Test [31,32], the frontal assessment battery (FAB) [33] and the IADL scale (IADL-E) [15] during the neuropsychological assessment.

### 2.2. Study Participants and Clinical Assessment in Thessaloniki in Greece

The protocol experiment in Thessaloniki included 98 participants (27 AD, 38 MCI, 33 healthy) aged 60–90. All participants were recruited at the Day Care Centre “Agia Eleni” of Greek Alzheimer Association in Thessaloniki, Greece. The diagnosis was given by the neurologist and the neuropsychological assessment was conducted by psychologists working in the Centre. The diagnosis of AD followed the criteria of the NINCDS-ADRDA and DSM-IV [34] and the Albert criteria of diagnosing MCI [35]. Each participant gave informed consent before the assessment (Table 1).

We administered a neuropsychological battery in order to assess the particular cognitive functions such as working memory, daily functionality, attention, memory, language and executive functioning. The instruments included the Greek version of Mini Mental State Examination (MMSE) [36], the Functional Rating Scale for Dementia (FRSSD) and the Functional Cognitive Assessment (FUCAS) [37]. Additionally, all participants were also assessed for depression with the geriatric depression scale (GDS) [38] and those who had high scores in the depression scale were excluded from the study.

### 2.3. Study Protocol in Both Sites

Based on the lab data analyses, we could show that Dem@care serves as an additional assessment tool improving the early detection of dementia, thus able to detect fine subtle behavioral changes in the different patient groups. We demonstrated with several sensor analyses studies that it is possible to obtain, just based on the sensor-extracted data, relatively high accuracy rates to differentiate between healthy, MCI and AD subjects.

The different studies performed using the same protocols in both Thessaloniki and Nice were highly innovative, and were among the first ones to try to demonstrate the use of ICT-based tools for clinical assessment purposes of dementia patients. The aim was to validate the sensor measurements by associations with classical assessment instruments and accordingly promote a holistic solution for the remote management of people with dementia. From the early beginning of the project, patients were involved in the co-design process of the multiple sensor-based system, for example by taking into account the acceptability of various sensors. Several advances in challenging problems in visual sensing were made to serve the goals and purposes of the Dem@Care system. Video data collected from wearable and static sensors were calibrated and fused to take advantage of their complementary nature. This leads to improved activity recognition performance, thanks to additional localization information that provides context to the other camera data. Person detection and tracking methods were developed that make use of the contextual scene information for accurate person localisation and tracking.

A comprehensive view of the patient’s lifestyle, behavioral patterns and daily activities was studied for accurate diagnosis, and for correlating observed behaviors with the different stages of dementia. This will significantly advance the typical clinical workflow for dealing with dementia, which currently involves very subjective and incomplete means of recording, such as questionnaires and diaries.

The main goal of the evaluation was to assess whether the Dem@Care system could contribute to the conventional assessment methods and procedures for the diagnosis of cognitive and neuropsychiatric symptoms. In addition, the ability of people with dementia to perform activities of daily living was also assessed. More specifically, the lab-based assessment was developed in order to gain a better knowledge of the clinical characteristics and the behavior of people with AD and their interaction with ICT while performing activities of daily functioning. The lab-based test and evaluation and the research connected to it is primarily concerned with the assessment and diagnoses of people with dementia. Therefore, we tested individuals with the diagnosis of early AD, MCI and compared their results with the HC so as to assess the effectiveness and the clinician usability of the Dem@Care system. The evaluation was based on gathering data from different sensors, in combination with video data, while participants were performing the clinical protocol. The main focus was on the assessment of the functioning in the instrumental activities of daily living and data from conventional clinical assessments.

The Thessaloniki scenario was the same as the Nice scenario, with minor changes in each task (e.g., position of the items) and additional sensors. The goals of the protocol were (a) to support clinicians in the assessment of autonomy and functionality in daily activities of dementia patients, (b) to investigate the accuracy and the effectiveness of the system. We selected specific activities for the evaluation which were found to be particularly deteriorated in the AD spectrum and specifically in the preclinical stages since they constitute complex ADLs and engage several cognitive functions (e.g., executive function, financial capacity, medication treatment) instead of simple ADL activities which play a pivotal role in more advance stages (e.g., use of the bathroom, preparing the bed, getting on and off a seat). There are two types ADLs than can be distinguished [39]. The first category is called “Basic ADL” and includes activities such as eating, bathing, dressing up, and mobility, which is considered to be widely preserved in MCI population. On the other hand, IADLs are usually deteriorated in the course of cognitive decline [40]. In particular, IADLs incorporate activities, for instance, related to shopping, meal preparation, managing finance, using the telephone and transportation and taking of medication, and other several daily activities that engage complex cognitive functions [41]. Therefore, in the lab assessment, we included “Semi-directed” IADLs since we examined people with MCI and mild to moderate AD, in order to assess the autonomy of the participants and their performance in ADLs. The participant had to correctly perform a list of daily tasks within a timeframe of 8 min. For this step, the participant was alone in the experimental setting and could refer to the instruction sheet at any time. The clinical protocol contained the following activities:

- Prepare drink (e.g., tea);
- Make a phone call to a specific number;
- Establish account balance and transfer money through a tablet device to a specific account;
- Prepare drug box following a prescription.

**Figure 1:**
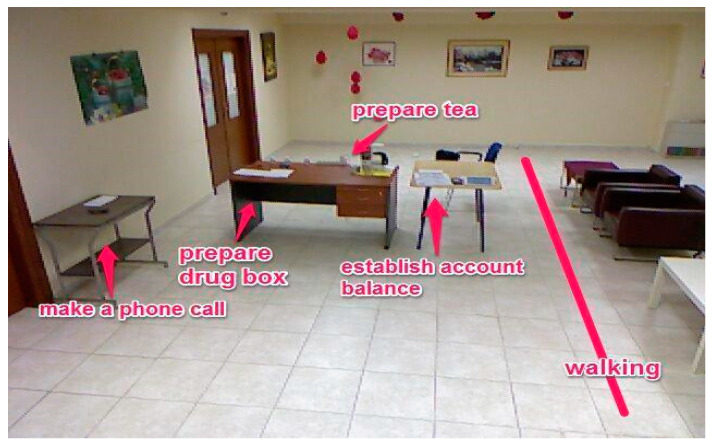
Thessaloniki short protocol installation.

**Figure 2:**
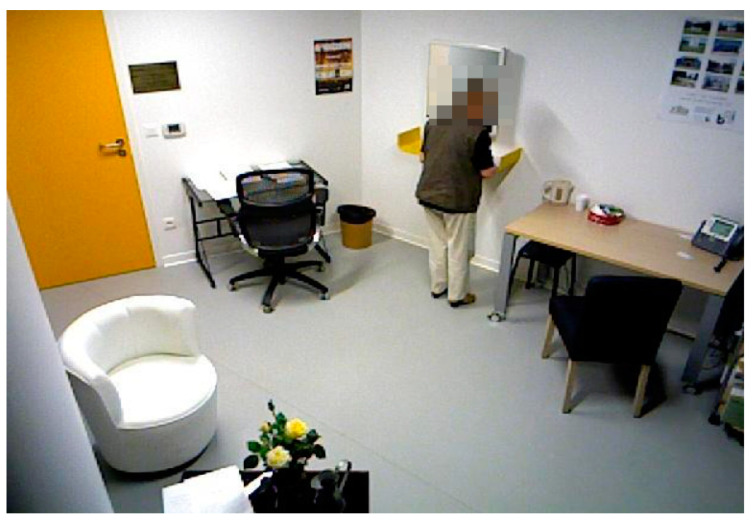
Nice protocol installation.

**Figure 3:**
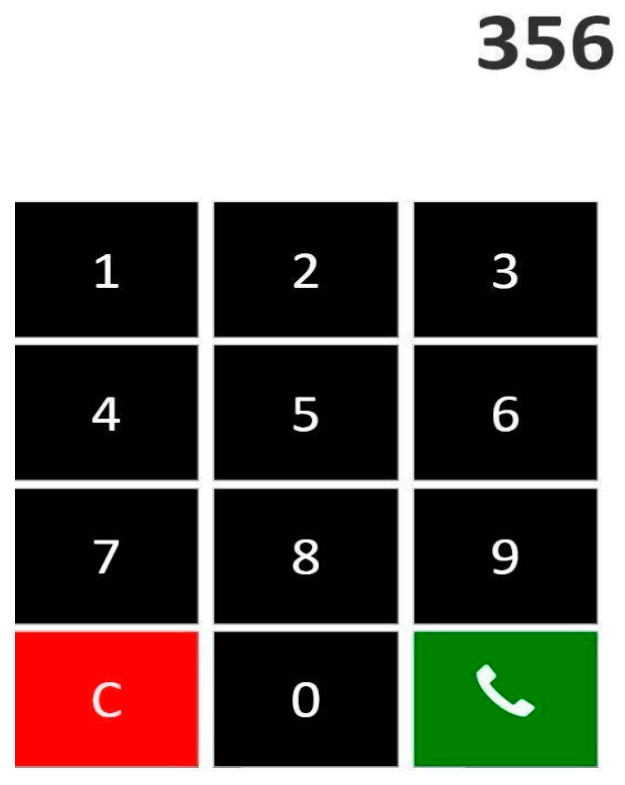
Phone app.

**Figure 4:**
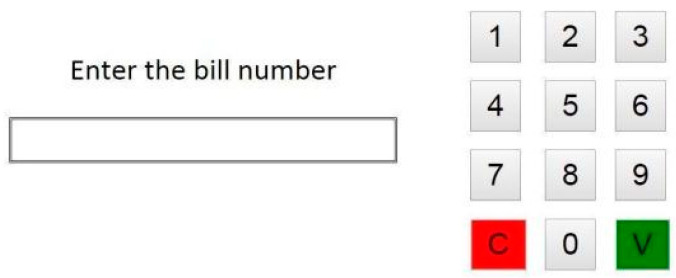
Money transfer app.

The installation followed the same principles in both pilot sites. We tried to have identical objects and distances between activities (Figure 1; Figure 2). In two activities (establish account balance and make a phone call), the participants used apps in mobile devices to accomplish them. The first app simulated a phone operation and it was used in a smartphone to record various elements of the ‘Make a phone call’ task (e.g., correct number) (Figure 3). The second app simulated a bank account transference and it was used in a tablet (Figure 4).

#### 2.3.1. Complex Activity Recognition System

To objectively evaluate the performance of participants during gait and IADL tasks, we employed the Complex Activity Recognition (CAR) system [27,42]. In particular, it takes as input a recording of the scene by a 3D camera (ASUS Xtion Pro Live). For each participant, the system analyzes the respective video recording and automatically recognizes the beginning and ending of the IADLs carried out during the semi-directed activities scenario.

The CAR system is composed of four main modules: people detection, people tracking, gait analysis and event recognition. People detection is performed by the background-subtraction algorithm proposed by a recent study [43]. The people that are detected in the scene are detected in specific time and space by using the algorithm proposed by [44], while the output of these two units is afterwards used as an input for “gait analysis” and “event recognition”. Event recognition is based on [42], where a constraint-based ontology language is employed to model activities of daily living, given the posture, motion and scene location patterns automatically extracted by underlying modules.

**Figure 5:**
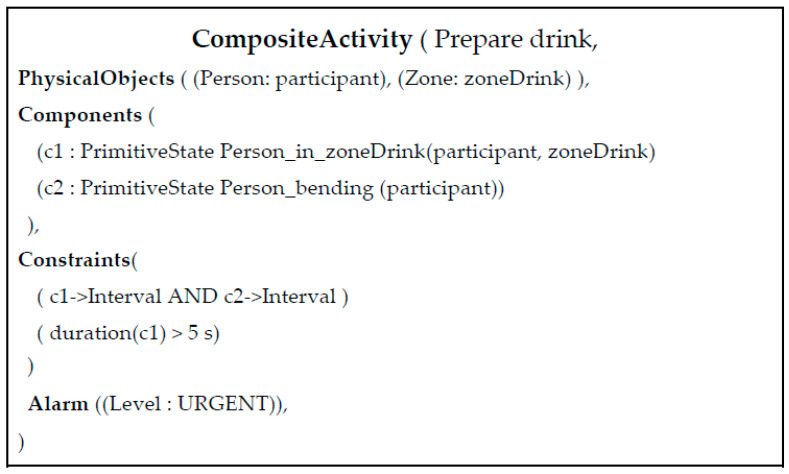
Composite activity model for the “Prepare drink” activity.

An IADL model consists of the enumeration of physical objects (e.g., detected people, room furniture and objects) and sub-events that intervene in the targeted IADL. Sub-events are intermediate activities (steps) that a person realizes to accomplish a more complicated task, in this case an IADL. An IADL model also contains constraints that define rules over sub-events and physical objects, like the order that two sub-events should appear in time to be a valid instance of the targeted activity. Figure 5 presents the event model of the “Prepare Drink” activity. This model has two sub-events (components): one sub-event that verifies whether the person’s global position is located in the spatial location where drinking objects are generally placed (named Person_in_zone_Drink), and a second sub-event verifying whether the person displays the posture “bending” (named Person_bending). The first constraint (or rule) of the model defines that both sub-events must be performed by the person at the same time (c1->Interval AND c2->Interval). The second constraint establishes that the sub-event “Person_in_zoneDrink” must have been performed by the participant for at least 5 s to characterize a “Prepare drink” event. Once both constraints are satisfied, the event starts to be recognized by the CAR. A more detailed description of the IADL analysis can be found in our recent study [42]. In detail, Figure 5 illustrates the monitored scene annotated with the semantic data deployed for event recognition and modeling, whereas the left picture image shows the “preparing tea” event.

At the end of the analysis of a video, the CAR system generates a report that constitutes the basis for the computation of the performance of the patients in the clinical protocol. The report contains the frequency and duration of each type of IADL carried out by a participant during an evaluation session, as well as the number of times these have been missed or repeated. Repetitions and omissions of an activity are calculated with respect to the number of times participants are expected to perform them given the instructions they have received at the beginning of the experiment.

#### 2.3.2. Statistical Analysis

Statistical analysis was performed using SPSS software, version 23. Analyses included the chi-square test, the Wilcoxon–Mann–Whitney test, one-way analysis of variance (ANOVA), mixed between/within subjects ANOVA and correlation analyses.

#### 2.3.3. Results

##### Population in Nice

Previous studies involving Nice senior population have shown the interest of using automatic video analysis as an additional source of information for IADL assessment in dementia patients [10,27]. In prior work, participants were asked to carry out physical tasks and a set of seven IADLs. In order to possibly integrate this method into daily clinical practice, a shorter version of the protocol was designed, focusing on the four above listed IADLs. The implementation of the short protocol in Nice was a preliminary evaluation/study with a small number of participants prior to the implementation of the short protocol with a larger number of participants in Thessaloniki. In Table 1, the demographic characteristics of the participants in Nice are presented.

The group in Nice included a total of 11 participants, of which 6 individuals were diagnosed with MCI (mean age = 75.83 ± 5.95, MMSE = 27 ± 2.68) and 5 were healthy controls (mean age = 71.6 ± 2.51, MMSE = 28.6 ± 0.55). There was no significant difference between the three groups in gender (X^2^ = 2.40, *p* = 0.12), age (*p* = 0.31) or education (*p* = 0.91). Individuals diagnosed with MCI had a lower MMSE, FAB and Grober–Buschke score than the healthy control subjects.

##### Population in Thessaloniki

Cognitive assessment was performed by means of a neuropsychological test battery, which was the same with the short protocol. Table 2 shows the demographic characteristics of the participants in Thessaloniki. Ninety-eight participants were included in the study, of which 27 were diagnosed with AD, 38 with MCI and 33 were HC. No statistically significant difference was found among the three groups with regards to gender (X^2^ (2,67) = 3.63, *p* = 0.163), education or age (F (2,66) = 1.63, *p* = 0.204). As expected, AD participants had a statistically significantly lower total score of MMSE compared to the participants with MCI and HC, while the MCI participants had a lower MMSE with regards to HC (Table 3). The differences between the HC and MCI are rather small and seem to be only in cognition and memory, and not in functionality or activities of everyday living.

#### 2.3.4. Statistical Analysis Based on CAR Results and Ground-Truth Data

In the following table (Table 4), the statistical analysis (ANOVA, multiple comparisons) based on the CAR analysis is presented. From the CAR activity report, the analysis showed the statistically significance differences between the MCI and AD participants in the following protocol activities: (1) Make payment duration and (2) Talk on the phone duration.

Moreover, we conducted a paired sample t-test of all the participants regarding the duration of the talk in the phone activity. The results were also statistically significant (*p* = 0.378).

Finally, in the following table (Table 5), correlations between the activities, age, education and MMSE are presented.

#### 2.3.5. Comparison of Two Sites

**Figure 6:**
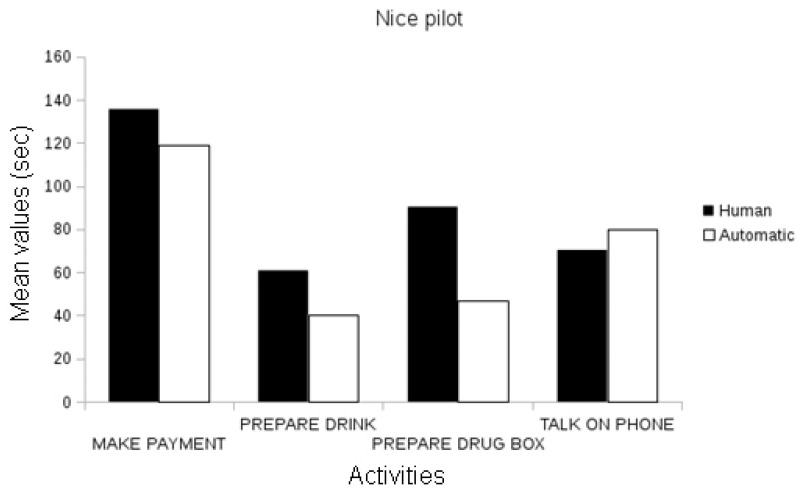
Comparison between the assessed duration (in seconds) of the automatically recognized events and the ground-truth data in the Nice Pilot.

The first evaluation conducted validated the measurement of the event recognition system compared to the ground-truth data, i.e., the events observed and annotated by domain experts (e.g., clinicians) per pilot site. In the Nice pilot, the events annotated by domain experts automatically recognized by an event recognition system were statistically correlated in duration with the duration of events manually annotated (Pearson’s r, *p* < 0.01; see Figure 6).

**Figure 7:**
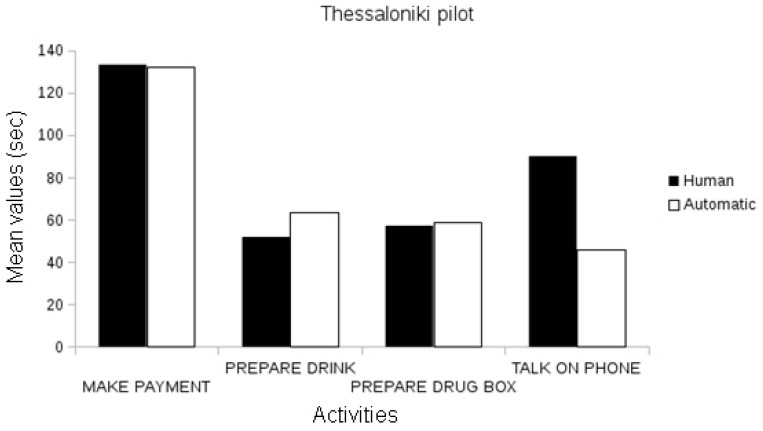
Comparison between the assessed duration (in seconds) of automatically recognized events and ground-truth data in the Thessaloniki Pilot.

In the Thessaloniki pilot, automatic event recognition was statistically correlated with the annotated events, both in frequency and in the duration attributes for all activities (Pearson’s r, *p* < 0.01), with the exception of the frequency parameter of preparing the drug box and the talk on the phone events, which were marginally correlated (Figure 7).

Given these results, we may conclude that the automatic event recognition system provides event measurements correlated to events manually annotated by domain experts. We observe that the correlations between manually annotated events and automatically extracted events increase with the number of participants. For instance, the analysis of the short protocol in the Nice pilot contains 11 participants and has fewer correlations between the extracted event and ground-truth annotations than Thessaloniki event data, which is composed of 98 participants.

##### Comparison between Cognitive Status Groups

The second evaluation concentrated on the information derived from the activities performed by the participants of the laboratory pilots and tests, for the statistical differences in these activities between the different cognitive status classes (memory cognitive impairment—MCI, Alzheimer, and healthy).

**Figure 8:**
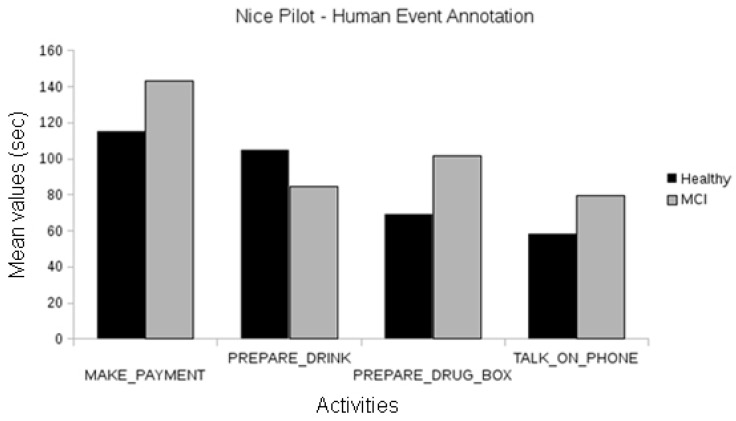
Comparison between the duration of manually annotated events of the different cognitive status groups of the Nice pilot.

**Figure 9:**
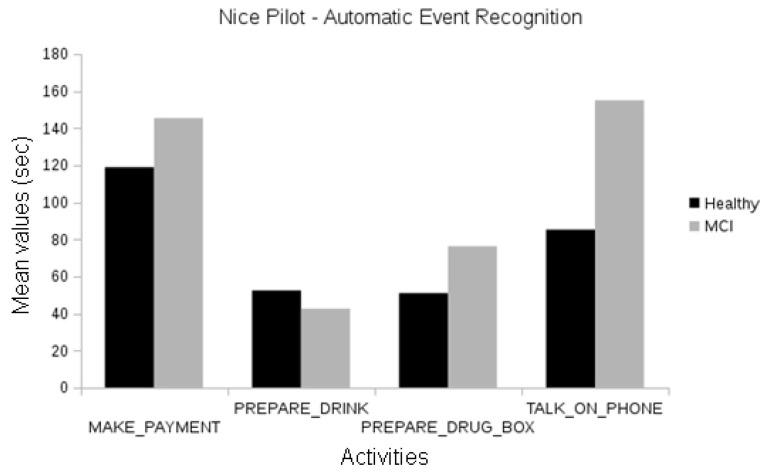
Comparison between the duration of automatically recognized events of the different cognitive status groups of the Nice pilot.

In the Nice pilot, no statistically significant differences were found between the MCI and the healthy participants, neither using human annotations of events (Figure 8) nor automatically recognized events (Figure 9). Since this evaluation focused on the short version of the laboratory protocol, there were not enough participants for a significant comparison between the Alzheimer’s group and the others.

**Figure 10:**
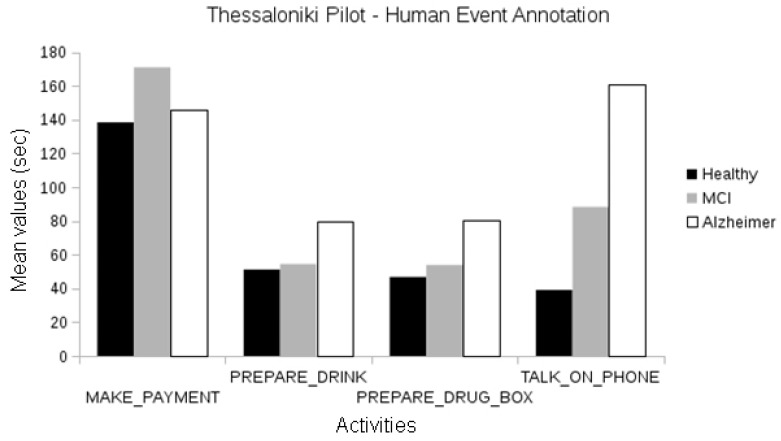
Comparison between the duration of manually annotated events of the different cognitive status groups of the Thessaloniki pilot.

In the Thessaloniki pilot, when analyzing events manually annotated by human experts, we observed differences between the duration of activities among cognitive classes for the “talk on the telephone” event between the healthy and Alzheimer groups and the healthy and MCI groups (one-way ANOVA, *p* < 0.01). Differences in the duration of “make payment” events were also observed between the healthy and Alzheimer participants and MCI and Alzheimer participants (one-way ANOVA, *p* < 0.01; see Figure 10).

**Figure 11:**
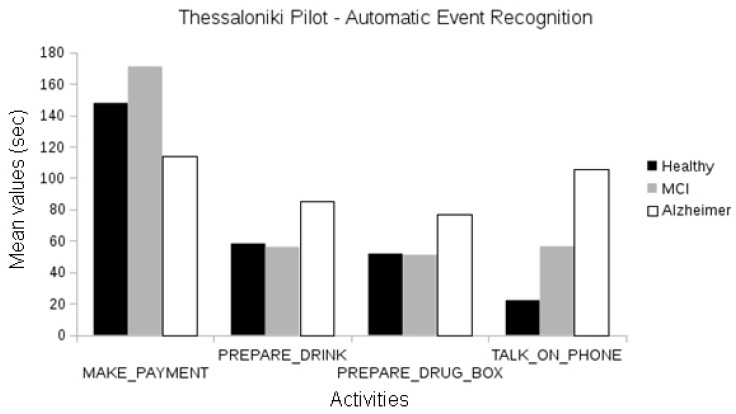
Comparison between the duration of manually annotated events of the different cognitive status groups of the Thessaloniki pilot.

When using automatically recognized events, we observed statistically significant differences between the activities of healthy and MCI groups (frequency of “make payment”, duration of “talk on the telephone”; one-way ANOVA, *p* < 0.05). Differences in the duration of “make payment” activity are also observed for the healthy and Alzheimer participants (ANOVA, *p* < 0.05), and MCI and Alzheimer participants (*p* < 0.01). Gait-related events like walk and walking test second attempt also present statistically significant differences between the MCI and Alzheimer groups (ANOVA, *p* < 0.05); see Figure 11.

In summary, we may conclude that the event recognition system results are accurate enough to reproduce the trends observed in the ground-truth data (e.g., statistical differences in the duration of the talk on the telephone and make payment events). Although there are certain events that highlight differences between cognitive classes (e.g., make payment event), there is no single parameter (e.g., event frequency) or activity that can discriminate all the classes of cognitive status.

##### Comparison between Laboratory Pilot Sites

In the third evaluation, we sought for differences between the activities of patients of different pilot sites (Nice, Thessaloniki) but same cognitive status (HC, MCI). For instance, would the healthy groups be different (in duration and frequency of activities) between the Nice and Thessaloniki pilot participants? We compared the Nice and Thessaloniki participants using the four usual instrumental activities of daily living with manually annotated IADL and the automatic recognition of IADL and gait-related events.

**Figure 12:**
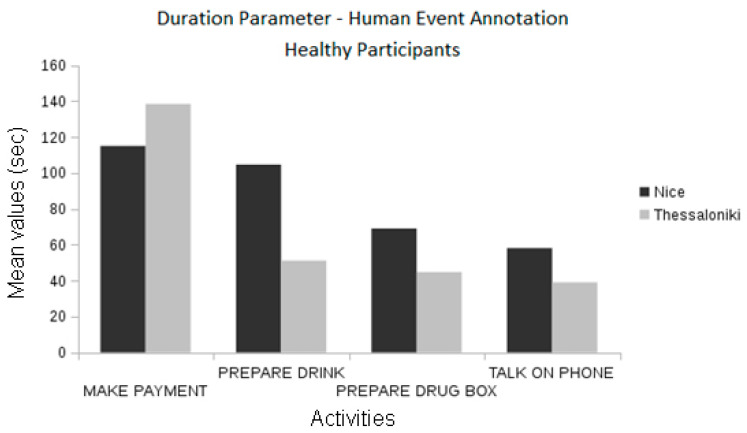
Comparison between the pilots concerning the event duration using the manually annotated data.

By comparing the event information from the annotations produced by domain experts, we found statistically significant differences between the healthy participants of the two pilots in the frequency of the “make payment” event and the duration of the “prepare drug box” event (ANOVA, *p* < 0.01 and *p* < 0.05, respectively, Figure 12). Differences in the “make payment” events are also observed between MCI groups (ANOVA, *p* < 0.01, see Figure 12).

**Figure 13:**
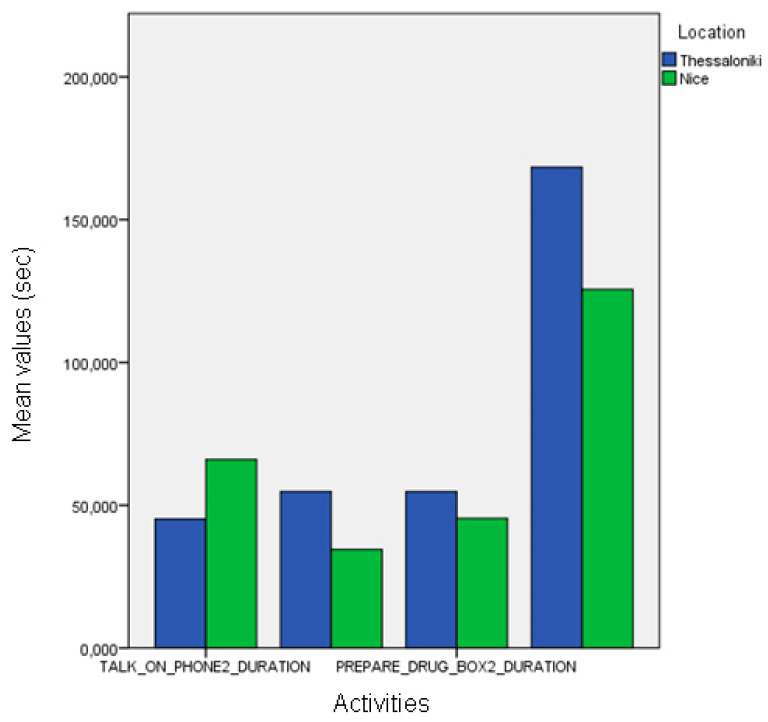
Comparison between the duration of the manually annotated events of different cognitive status groups of the Nice pilot.

**Figure 14:**
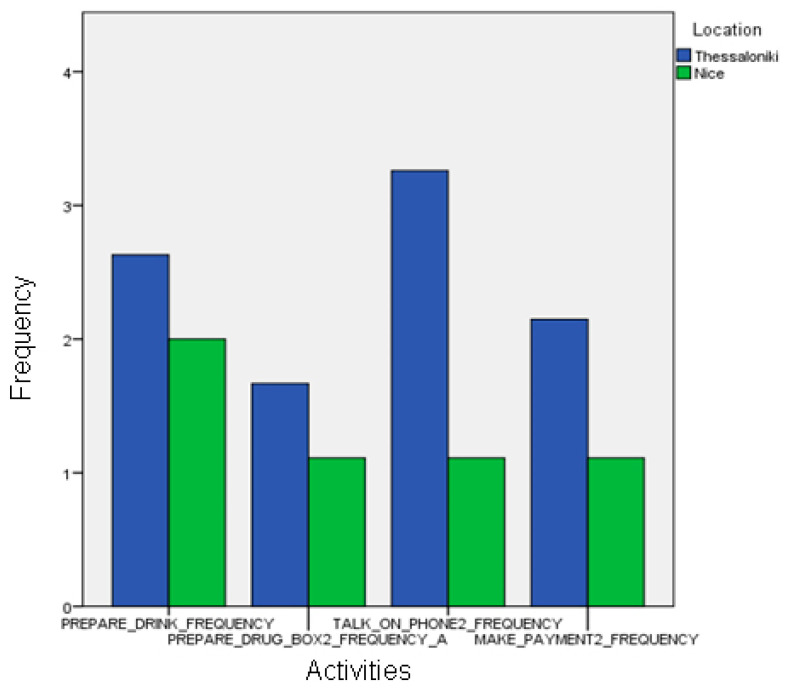
Comparison between the frequencies of the automatic event recognition between the two sites.

**Figure 15:**
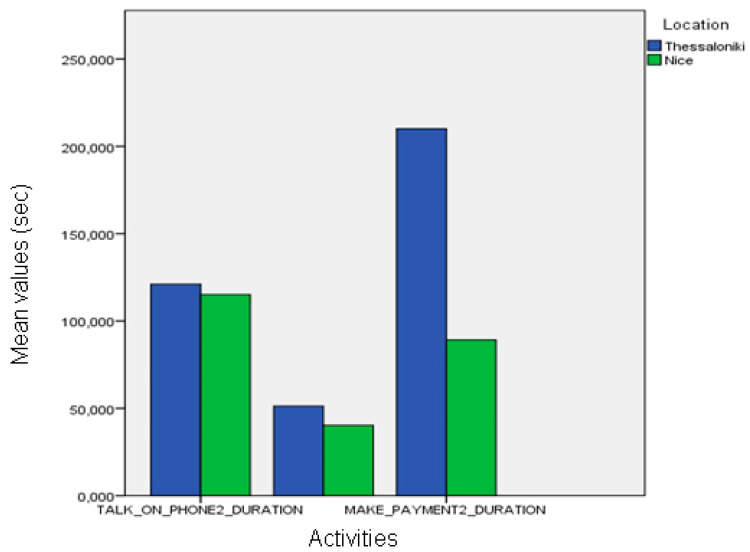
Comparison between the duration of automatic recognition between the two sites.

When automatic event recognition was used to analyze the performance of pilot participants we found that no statistically significant differences existed between the healthy participants of the Nice and Thessaloniki pilots (Figure 13), which was also observable in the ground-truth data. However, the differences found in the event frequency of “make payment” according to the ground-truth data were only marginally significant when using automatically extracted information (Figure 14). Nevertheless, there are statistically significant differences between the duration of “talk on the telephone” event (ANOVA, *p* < 0.01) both between healthy participant groups and between MCI groups (Figure 15). The latter differences may be a fine pattern not observable before due to the subjective component of the manual annotation of events, and will be object of study in further work.

## 3. Discussion

The evaluation process of the present study has been conducted in the controlled environment of a clinical lab where controlled assessment procedures could be maintained which contribute to the possibility of generalizing the results. It addresses the need of improving existing diagnosing procedures of dementia and the related disorders by aiming at providing innovative and novel solutions for providing additional information for the assessment of specific domains (behavior, cognition, activity of daily living). This information, together with other clinical and biological data, can contribute to earlier and more accurate diagnosis procedures of AD and the related disorders. Early and timely diagnosis of dementia is an important aspect of improving the situation for people with dementia in early stages of the disease, by the early introduction of proper medical treatment and personalized support aimed at improving the ability to manage everyday life. Implementing new tools that are able to detect fine and subtle changes in behavioral, cognitive and functional patterns may allow earlier diagnosis, even at the point when memory functions are still intact. This could lead to earlier, and thus potentially a more effective prevention and treatment of AD. In this sense, the aim of this thesis was to investigate the possibility of using technologies for assessment purposes. The results of these studies led to the implementation of their use for outcome measurements within clinical trials in AD and related disorders.

ICTs can indeed provide useful information for assessing the specific domains of AD patient’s life, and hence address the current need to use innovative measures that demonstrate clinically meaningful cognitive, behavioral and functional outcomes. In addition, it is proposed to consider the use of ICT in the design of clinical trials. It is important to underline, that not one composite measurement alone can cover the entire spectrum of AD, from early to late stages. However, in combination with already existing clinical assessments and biomarkers, ICT can provide additional diagnostic relevant information that are captured in a more reliable and objective manner, and therefore complete the evaluation of a patient’s cognitive and functional status.

In order to integrate ICT measurements into large clinical cohort trials, some further research requires performing, namely the validation of the use of such technologies in larger cohorts to demonstrate clinical meaningfulness and thus, receive recognition in the clinical scientific and medical world. This could eventually lead to a change of attitude in general practitioners and research investigators towards more willingness for using ICT in routine assessment procedures. The ‘de-mystification’ of ICT usage by showing that it is actually easy and simple to use could facilitate its gradual integration in the users work routine and increase acceptability.

Based on the fact that ICT methods have been criticized, firstly for being still strongly dependent on a human observer; and secondly for removing the individual’s chosen routine and environmental cues that typically facilitate IADL, the central idea of this paper was to investigate whether the same protocol of these studies with the same equipment can be successfully transferred to another clinical site, in another country with a different cultural context, and still provide comparative results for each country. The experimental results suggest that sensor data collected in an ambient assisted living environment can be used to assess task quality (resulting from the automatic detection of task duration and frequency) and provide clinicians with results which are correlated with standardized validated tests.

The results of the short protocol pilot study revealed that the healthy participants outperformed the MCI, who outperformed the AD participants. This observation was only possible to capture due to the use of the automatic video analysis system during the protocol. The ANOVA test has shown statistically significant results in almost every task characteristic which could mean that using such protocol and tools could both improve the assessment of IADL functioning and also assist in detecting markers for prodromal stages of dementia (Table 4).

Nowadays, the majority of clinicians use specific neuropsychological tests in order to perform an accurate diagnosis of a patient, but also to discriminate prodromal stages of dementia from mild cognitive impairment stages and from elders with no memory and cognitive deficits. However, the majority of clinicians are divided with regard to scoring and subjective assessment. In our assessment, the objective measurement of these patients is a clue for their diagnosis in correlation with their performance in neuropsychological testing. Moreover, in the Thessaloniki data set, we applied three specific measurements: MMSE, about general cognitive status, and FUCAS, regarding the information about everyday functionality and FRSSD, about functionality. We found that there are strong correlations between these measurements and the participants’ performance in IADLs, according to the automated assessment. These results indicate that even people with MCI can be discriminated from patients with mild cognitive impairment and healthy groups via a short-time, objective and accurate assessment.

Furthermore, our results indicated that there is a strong correlation between the tasks of preparing tea and preparing a drug box. This was an expected result as these two tasks were the most complex ones, since they included various steps. On the other hand, the sensors seem more sensitive and efficient in order to predict between the MCI and healthy participants. Results are also statistically significant for the differences between MCI–AD and healthy–AD groups.

In a nutshell, in this cross-pilot evaluation, we firstly demonstrated that the CAR system provides activity recognition data for the analyses of the performance of pilot participants’ IADL activities. Therefore, the proposed system enables the objective assessment of patient performance data with relatively less effort than the manual annotation of activities by human experts without the common observer biases. Secondly, we observed that certain activities and derived parameters may discriminate participants from certain groups of cognitive status (make payment event), but no parameter can discriminate all cognitive classes. In this sense, as discussed in [10,27], more sophisticated models are necessary to model the differences between the cognitive status classes (healthy participants versus MCI; and MCI participants versus Alzheimer’s participants) and support clinicians by providing them with an objective assessment regarding the patient’s cognitive status.

Consequently, the objective of this study was to investigate whether the discussed ICT-based clinical protocol will provide similar results when applied to two different countries with a different cultural context. The experimental results suggest that automatic activity recognition output data can be used to assess IADL task quality and provide clinicians with quantitative results which correlate with standardized, objective and commonly used neuropsychological tests. Moreover, the present study has also demonstrated that the proposed short clinical protocol can be successfully applied in different clinical sites, e.g., Nice (France) and Thessaloniki (Greece), remaining effective on its statistical power to highlight the differences between the performances of participants of different cognitive states. Introducing new tools, which were able to detect fine and subtle changes in behavioral, cognitive and functional patterns, may allow earlier clinical attention even at the point of a pre-dementia state (e.g., MCI). This could lead to the timely and thus potentially more effective prevention and treatment of AD and other variants of dementia. In this sense, the aim of this evaluation was to investigate the use of ICT technologies for assessment purposes. ICTs can indeed provide useful information for assessing specific domains of a patient’s life, and hence address the current need for new innovative measures of clinically meaningful cognitive, behavioral and functional changes. It is important to underline that no single composite measurement alone can cover the entire spectrum of AD, from early to late stages, and as a result, no consensus exists as to which is the best tool to categorize the participants who face cognitive disturbances. However, in combination with already existing clinical assessments and biomarkers, ICTs can provide additional information for diagnostic purposes that is captured in a more reliable and objective manner, and therefore completes the evaluation of a patient’s cognitive and functional status.

To integrate ICT measurements into large clinical cohort trials, some research still has to be done, namely the validation of the use of such technologies in larger cohorts to demonstrate clinical meaningfulness and thus, receive recognition in the medical world. This could eventually lead to a change of the attitudes of general practitioners and research investigators towards more willingness for using ICTs in routine assessment procedures. For instance, during the initial visit of the patient, where the neuropsychological, neurological and any additional examinations which are taken place to determine the diagnosis, the lab examination could be considered as an additional examination. The ‘de-mystification’ of ICT usage by showing that it is actually possible to use, could facilitate its gradual integration into normal clinical work practice and increase its acceptability among clinicians. Furthermore, in future research, the possibility of using such tools to predict dementia stages and disease outcome should be investigated through longitudinal studies of monitoring healthy older adults by the means of ICT over time. This work revealed that the proposed ICT solution will work successfully in two different sites with different cultural aspects.

The present study show that the Dem@care framework and the assessment of the daily activities through a lab-simulated home environment constitute a significant tool for objective assessment, as well as timely and accurate diagnosis, by improving the understanding of how the AD and its preclinical stages affect the patients’ daily activity and cognition. In particular, it constitutes a multi-parametric closed-loop remote monitoring solution which enables clinicians to obtain a comprehensive view of the patients and their daily difficulties. Finally, the paper proved that the short protocol was successfully transferred and implemented in Thessaloniki with very encouraging and significant outcomes, which could be successfully implemented in other populations with no particular educational or cultural background.

This study demonstrated the feasibility of such innovative ecological assessment, including the protocol design and the technical set up, as well as its transferability from one clinic to another one in two very different cultural contexts. We emphasize the cross-site comparison to underline the importance of developing tools that can be widely used across different clinical settings. This is relevant for the community since it shows how easily a common standardized measure of autonomy could be implemented in clinical practice. Until now, the way IADLs are evaluated is rather limited and not always representative of the reality of what a patient is still able to do at home, therefore we need to have better and more objective assessment tools. Our study shows that with the help of new technologies, we can provide information about a patients’ autonomy level in a completely automatized way—this means that in our scenario, more timely intervention could be provided when decline is detected.

## Figures and Tables

**Table 1:** Demographic characteristics of Nice pilot’s participants.

	MCI (*n* = 6) M(SD)	Healthy (*n* = 5) M(SD)	*p*
Sex	Women 2 Men 4	Women 4 Men 1	*p* = 0.12
Age	75.83 (5.95)	71.6 (2.51)	*p* = 0.31
Education (in years)	11.33 (3.83)	11.25 (2.87)	*p* = 0.91
MMSE Lawton Scale	27 (2.68)	28.6 (0.55)	*p* = 0.28
FAB Lawton Scale	14.4 (0.55)	17 (1.41)	*p* = 0.017
Cued Selective Reminding Test Grober–Buschke Overall total score (first, second and third free recall trial)	34.75 (11.79)	47.25 (0.5)	*p* = 0.027
IADL Lawton Scale	6.5 (1.29)	7.4 (0.55)	*p* =0.30

**Table 2:** Demographic characteristics of Thessaloniki pilot’s participants.

	MCI (*n* = 38) M(SD)	AD (*n* = 27) M(SD)	Healthy (*n* = 33) M(SD)	*p*
Sex	Women 28 Men 10	Women 22 Men 5	Women 21 Men 12	*p* = 0.16
Age	69.811 (5.8634)	73.333 (6.8219)	65.800 (3.9397)	*p* = 0.00
Education Years	11.919 (3.9397)	9.926 (4.4021)	12.300 (3.8788)	*p* = 0.07

**Table 3:** Comparison between the patients with MCI, the patients with Alzheimer’s disease (AD) and the healthy controls.

	AD		MCI		Healthy		
	Mean	Std. Deviation	Mean	Std. Deviation	Mean	Std. Deviation	*p*
MMSE	21.074	4.4021	27.703	1.8539	28.968	1.0796	*p* < 0.001
FRSSD total score	9.815	4.1606	3.919	1.8315	2.323	1.7774	*p* < 0.001
FUCAS total score	57.815	12.9557	44.270	2.0636	42.118	0.4777	*p* < 0.001
FUCAS Medication	10.038	2.5057	7.324	0.7474	7.065	0.3592	*p* < 0.001
FUCAS Telephone	11.962	2.5843	8.027	1.0926	7.065	0.3592	*p* < 0.001
FUCAS Shopping	9.654	2.5914	7.486	0.9609	7.000	0.0000	*p* < 0.001
FUCAS Transport	10.154	2.8940	7.432	0.8347	7.065	0.3592	*p* < 0.001
FUCAS Memory	8.923	1.5472	6.595	0.7623	6.032	0.1796	*p* < 0.001
FUCAS Planning	7.846	1.9533	6.081	0.2767	6.000	0.0000	*p* < 0.001
FUCAS Time	7.115	1.5831	6.000	0.0000	6.000	0.0000	*p* < 0.001

**Table 4:** Statistical analysis based on the Complex Activity Recognition (CAR) output.

**ANOVA**		**Sum of Squares**	**df**	**Mean Square**		**F**	***p***
**Make Payment Duration**	Between groups	196,141.97	2	98,070.985		4.46	0.015
	Within groups	1,561,221.167	71	21,989.031			
	Total	1,757,363.137	73				
**Multiple Comparisons**		**Mean Difference (I–J)**			**Std. Error**		***p***
**Talk on Phone Duration**	Games–Howell	75.937287			29.561122		0.039
**Make Payment Duration**	Tukey HSD	−122.934106			41.600699		0.012

**Table 5:** Correlations between the activities, age, education and MMSE in Thessaloniki.

Correlations (Duration Attribute)	Age	Education	MMSE
Prepare Drug Box	0.215	−0.369 **	−0.149
Make Payment	−0.126	−0.104	0.248

** Correlation is significant at the 0.01 level.

## Abbreviations

AD	Alzheimer’s Disease
MCI	Mild Cognitive Impairment
IADL	Instrumental Activities of Daily Living
DAD	Disability Assessment for Dementia scale
FLSA	Functional Living Skills Assessment
ICT	Information Communication Technologies
AAL	Ambient Assisted Living
MMSE	Mini-Mental State Examination
HC	Healthy controls
FAB	Frontal Assessment Battery
FRSSD	Functional Rating Scale for Dementia
FUCAS	Functional Cognitive Assessment
GDS	Geriatric Depression Scale
CAR	Complex Activity Recognition
